# Mental Health Status and Healthcare Utilization Inequity Among People Living With HIV: A Cross‐Sectional Study Using Inverse Probability Treatment Weighting

**DOI:** 10.1155/da/4192199

**Published:** 2026-07-07

**Authors:** Ji Ma, Ticheng Xiao, Hang Chen, Mengjie Li, Mingzhe Ding, Run Chen, Xiaoxue Chen, Yanhua Chen, Fuli Huang, Ailing Li, Song Fan

**Affiliations:** ^1^ Department of Social Medicine, School of Public Health, Southwest Medical University, Luzhou, Sichuan, China, swmu.edu.cn; ^2^ Department of Prevention and Health Care, Sichuan University Hospital, Chengdu, Sichuan, China; ^3^ Luzhou Center for Disease Control and Prevention, Luzhou, Sichuan, China; ^4^ School of Nursing, Southwest Medical University, Luzhou, Sichuan, China, swmu.edu.cn; ^5^ Department of Infectious Diseases, The Affiliated Hospital of Southwest Medical University, Luzhou, Sichuan, China, ahswmu.cn

**Keywords:** concentration index, health equity, healthcare utilization, HIV, inverse probability treatment weighting, mental health

## Abstract

**Background:**

Psychological distress is highly prevalent among people living with HIV (PLHIV) and may impact healthcare utilization patterns. However, the relationship between mental health status and healthcare utilization inequity in this population remains poorly understood, particularly in resource‐limited settings. This study aimed to examine the association between mental health status and healthcare utilization inequity among PLHIV using robust methodological approaches.

**Methods:**

This cross‐sectional study included 418 PLHIV receiving antiretroviral therapy (ART) at major HIV treatment centers in southwestern China. Mental health status was assessed using validated Chinese versions of the Patient Health Questionnaire‐9 (PHQ‐9) and Generalized Anxiety Disorder‐7 (GAD‐7) scales. Healthcare utilization measures included self‐rated health (SRH), 2‐week morbidity, chronic disease prevalence, and hospitalization rates. Inverse probability treatment weighting (IPTW) was employed to minimize confounding, and concentration indices (CIs) were calculated to assess healthcare utilization inequity. Decomposition analysis identified key contributors to observed inequities.

**Results:**

After IPTW adjustment, participants who were symptom‐positive (48.3%) demonstrated significantly poorer health outcomes compared to those who were symptom‐negative (51.7%). The symptom‐positive group showed higher rates of poor SRH (52.6% vs. 37.5%;*p* < 0.110), 2‐week morbidity (56.7% vs. 47.6%; *p* = 0.010), and chronic disease (49.0% vs. 41.6%;*p* = 0.030). Paradoxically, despite worse health status, this group had lower hospitalization rates (82.1% vs. 88.2%;*p* = 0.010). CIs revealed pro‐rich inequities across all outcomes, with particularly pronounced disparities in hospitalization (CI = −0.2379,). Decomposition analysis identified drug use history (−1.1×10^1^%, *p* < 0.001), physical activity patterns (1.4×10^0^%, *p* = 0.0099), and health record establishment (3.1×10^0^%, *p* < 0.001) as key contributors to healthcare utilization inequities.

**Conclusions:**

PLHIV with symptom‐positive experience significant disparities in health outcomes and healthcare utilization, characterized by poorer health status but lower healthcare service utilization. While pro‐rich inequities exist across all outcomes, the patterns and magnitude of these inequities differ for symptom‐positive individuals compared to their counterparts. These findings highlight the need for integrated mental health and HIV services, systematic healthcare management, and targeted interventions to address healthcare barriers in this vulnerable population.

## 1. Introduction

The global HIV epidemic remains a significant public health challenge, with an estimated 39.9 million people living with HIV (PLHIV) worldwide by 2023 [1]. While antiretroviral therapy (ART) has transformed HIV into a manageable chronic condition and significantly increased life expectancy [2], PLHIV now face complex challenges beyond viral suppression, including a high burden of comorbid conditions. Elevated psychological distress, including symptoms of depression and anxiety, has emerged as a critical concern, with prevalence estimates among PLHIV substantially exceeding those in the general population [3].

The co‐occurrence of HIV and psychological distress presents unique challenges for healthcare systems. Mental health conditions can accelerate HIV disease progression through both direct physiological pathways and indirect behavioral mechanisms [4]. Individuals with them often demonstrate reduced ART adherence and increased risk‐taking behaviors [5]. Importantly, distress is also linked to altered patterns of healthcare utilization, including avoidance, delay, or inconsistent engagement with services [6]. A recent meta‐analysis of nine studies found that PLHIV with comorbid mental health conditions had 15% lower odds of achieving ART adherence [7].

Furthermore, healthcare utilization among PLHIV is characterized by significant inequities. These disparities in access and outcomes are typically more pronounced among individuals facing intersecting vulnerabilities, such as those with comorbid psychological distress [8, 9]. This relationship is often exacerbated by the dual stigma associated with HIV and mental health conditions, as well as by socioeconomic disadvantages [10]. In the Chinese context, while policies like the “Four Frees and One Care” initiative have reduced financial barriers to HIV treatment, and recent mental health reforms aim to integrate services into primary care, the specific mechanisms through which psychological distress interacts with these systemic factors to shape healthcare utilization equity remain underexplored.

Despite the growing recognition of these interrelated challenges, critical gaps persist in the literature. Existing studies have predominantly focused either on the mental health challenges of PLHIV [11–13] or on their general healthcare utilization patterns [14, 15] Additionally, methodological limitations, particularly in addressing confounding factors, have hampered the generation of robust evidence regarding this relationship [16].

The present study aims to address these research gaps by examining the association between psychological distress and healthcare utilization inequity among PLHIV in China, employing a robust methodology. Using inverse probability treatment weighting (IPTW) to minimize confounding, this study seeks to (1) quantify disparities in health outcomes and healthcare utilization between PLHIV with and without elevated psychological distress; (2) assess the degree of socioeconomic‐related inequity in healthcare utilization through concentration index (CI) analysis; and (3) identify key contributors to observed inequities through decomposition analysis. These findings aim to inform the development of integrated, targeted interventions to promote healthcare equity in this vulnerable population.

## 2. Methods

### 2.1. Study Design and Population

This cross‐sectional study was conducted between January and December 2023 at designated HIV treatment hospitals in Luzhou, China. Eligible participants were adults (≥18 years) living with HIV who were receiving ART and registered with the local Center for Disease Control and Prevention (CDC). Individuals with severe cognitive impairment or communication barriers that could affect survey completion were excluded. A total of 418 participants provided written informed consent and completed the survey.

### 2.2. Measures

#### 2.2.1. Mental Health Assessment

Depressive symptoms were assessed using the Patient Health Questionnaire‐9 (PHQ‐9), a 9‐item self‐report measure originally developed by Kroenke et al. [17] with a total score ranging from 0 to 27. The scale demonstrates good reliability and validity in general and clinical populations [17]. Anxiety symptoms were evaluated with the Generalized Anxiety Disorder‐7 (GAD‐7), developed by Spitzer et al. (2006). It is a 7‐item self‐report instrument with scores ranging from 0 to 21 and has been validated in large community samples, showing good psychometric properties [18]. The Chinese versions of both instruments demonstrated good reliability in HIV‐infected populations [19, 20].

#### 2.2.2. Healthcare Utilization Measures

Four healthcare utilization indicators were assessed:

Self‐rated health (SRH)—SRH is a widely used composite measure reflecting both subjective and objective health status and is a valid predictor of morbidity and mortality [21]. Respondents rated their health on a 5‐point scale (“excellent,” “very good,” “good,” “fair,” and “poor”). For analysis, responses were dichotomized into “good” (excellent/very good/good, coded as 0) and “poor” (fair/poor, coded as 1).

Two‐week morbidity: defined as any illness or injury within 2 weeks before the survey requiring medical consultation, self‐medication, or affecting daily activities.

Chronic disease prevalence: based on physician diagnosis within 6 months before the survey or ongoing management of previously diagnosed conditions with therapeutic measures.

Hospitalization rate: any inpatient admission during the year before the survey.

### 2.3. Covariates

The sociodemographic information collected included age, gender, education level, employment status, marital status, and monthly income. Healthcare‐related variables included insurance type, regular medical check‐ups, and knowledge of HIV‐related policies. Lifestyle factors (smoking, alcohol consumption, and physical activity) and HIV‐related clinical data were also collected.

### 2.4. Statistical Analysis

#### 2.4.1. IPTW

To address potential selection bias and confounding, IPTW was employed. Propensity scores were estimated using a generalized linear model that incorporated covariates from three levels: (1) individual characteristics (age, gender, years of education, marital status, and household registration type); (2) personal health (body mass index [BMI]); and (3) economic characteristics (occupation type and monthly income).

The exposure of interest was defined as screening positive on either the PHQ‐9 or GAD‐7 scale. Accordingly, the symptom‐positive group comprised individuals meeting this criterion, while the symptom‐negative group included those who screened within the normal range on both scales.

Based on the estimated propensity score (*p*) from the generalized linear model, IPTW weights were calculated as 1/*p* for the symptom‐positive group and 1/(1−*p*) for the symptom‐negative group. The resulting weights were applied to reweight the observed sample, and causal effect estimates were derived from the weighted data.

We calculated standardized mean differences (SMDs) for each weighted covariate to determine whether there was a significant difference. When the SMD was 0.1 or less after IPTW, the confounder was considered to have no between‐group difference [22, 23]. Outcomes were comparable when there were no between‐group differences in all covariates.

#### 2.4.2. Healthcare Utilization Inequity Analysis

To assess socioeconomic‐related inequity in healthcare utilization, CIs were calculated based on concentration curves [24]. The curve plots the cumulative proportion of the sample ranked by income (poorest to richest) against the cumulative proportion of each health outcome. The CI ranges from –1 to + 1, with negative values indicating pro‐rich inequity, positive values indicating pro‐poor inequity, and zero indicating no socioeconomic‐related inequality. Greater deviation of the curve from the line of equality reflects larger inequity.

Decomposition analysis was conducted to identify contributors to the observed inequities. For each outcome (SRH, 2‐week morbidity, chronic disease prevalence, and hospitalization), a probit regression model was fitted to estimate marginal effects of explanatory variables. These were used to decompose the CI into percentage contributions of each factor to the overall inequity [25].

### 2.5. Ethical Considerations

The study adhered to strict confidentiality protocols. All investigators signed confidentiality agreements with the local CDC. Personal identifiers were removed during data processing, and data access was restricted to the authorized research personnel. The study was conducted in accordance with the Declaration of Helsinki and was approved by the Biomedical Ethics Committee of Southwest Medical University (SWMUIRBTX‐202406‐0017).

## 3. Results

### 3.1. Study Population Characteristics

Of the 481 eligible individuals approached for this study, 418 provided valid responses (response rate: 86.9%). Based on the mental health screening using PHQ‐9 and GAD‐7, 202 participants (48.3%) met the criteria for mental health disorders (symptom‐positive group) and 216 (51.7%) did not (symptom‐negative group). The study population ranged in age from 18 to 90 years (mean age: 57.1 ± 15.7 years). Women comprised 32.2% of the symptom‐positive group and 27.8% of the symptom‐negative group.

An initial comparison of baseline characteristics revealed significant differences between groups (Table 1). The symptom‐negative group was significantly older (57.1 ± 15.7 vs. 50.1 ± 17.6 years, *p* < 0.001), had lower partnership rates (52.8% vs. 65.3%, *p* = 0.012), and fewer years of education (7.1 ± 5.1 vs. 8.6 ± 5.5 years, *p* = 0.006). Other characteristics, including gender distribution, residential location, BMI, and employment status, showed no significant differences (all *p* > 0.05).

**Table 1:** Baseline characteristics before and after IPTW.

Covariate	Pre‐IPTW (*n* = 418)				Post‐IPTW (*n* = 836)^a^			
	Symptom‐negative (*n* = 216)	Symptom‐positive (*n* = 202)	*p*	SMD	Symptom‐negative (*n* = 416)	Symptom‐positive(*n* = 420)	*p*	SMD
Gender								
Male	156 (72.2)	137 (67.8)	0.382	0.096	294 (70.7)	296 (70.5)	0.973	0.003
Female	60 (27.8)	65 (32.2)			122 (29.3)	124 (29.5)		
Age (X [S])	57.08 (15.7)	50.12 (17.6)	<0.001	0.417	54.05 (16.5)	54.13 (17.6)	0.962	0.005
Address (%)								
Rural	87 (40.3)	67 (33.2)	0.160	0.148	156 (37.5)	159 (37.9)	0.942	0.007
Urban	129 (59.7)	135 (66.8)	—	—	260 (62.5)	261 (62.1)	—	—
BMI (X [S])	22.09 (3.4)	21.58 (3.5)	0.129	0.149	21.86 (3.3)	21.87 (3.7)	0.990	0.001
Partner (%)								
Yes	114 (52.8)	132 (65.3)	0.012	0.258	244 (58.7)	247 (58.8)	0.975	0.003
No	102 (47.2)	70 (34.7)	—	—	172 (41.3)	173 (41.2)	—	—
Work (%)								
Student	2 (0.9)	5 (2.6)	0.281	0.222	7 (1.7)	7 (1.7)	1.000	0.008
Employed	95 (44.0)	91 (45.0)	—	—	185 (44.5)	188 (44.8)	—	—
Retired	33 (15.3)	19 (9.4)	—	—	51 (12.3)	51 (12.1)	—	—
Jobless	11 (5.1)	14 (6.9)	—	—	24 (5.8)	25 (5.9)	—	—
Unemployed	75 (34.7)	73 (36.1)	—	—	149 (35.7)	149 (35.5)	—	—
Years of education (X [S])	7.1 (5.1)	8.6 (5.5)	0.006	0.271	7.8 (5.3)	7.8 (5.5)	0.983	0.002

^a^The sample size in question pertains to the adjustment of the overall population sample size using IPTW, thus it is regarded as a “pseudo‐sample size.”

### 3.2. Balance Assessment After IPTW

**Figure 1:**
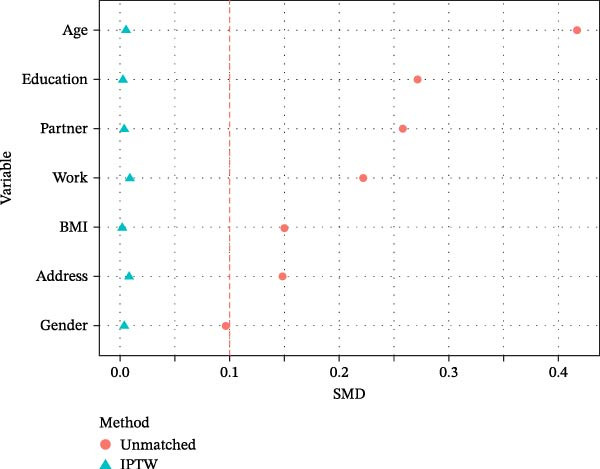
Standardized mean differences before and after IPTW.

Following IPTW application, all SMDs were reduced to less than 0.1, indicating successful balance achievement (Figure 1). The weighted analysis included 836 pseudo‐observations (417 symptom‐negative and 419 symptom‐positive), with no significant differences remaining in measured covariates (all *p* > 0.90, Table 1).

### 3.3. Health Outcomes Analysis

After IPTW adjustment, significant disparities emerged in all health outcomes between groups (Table 2). The symptom‐positive group consistently demonstrated a poorer health status across multiple indicators. Notably, a higher proportion of the symptom‐positive group rated their health as poor compared to the symptom‐negative group (52.6% vs. 37.5%; *p* < 0.001). Similarly, the symptom‐positive group reported significantly higher recent illness rates, with 2‐week morbidity reaching 56.7% compared to 47.6% in the symptom‐negative group (*p* = 0.010). Chronic disease prevalence also showed a similar pattern, with 49.0% of the symptom‐positive group reporting chronic conditions compared to 41.6% in the symptom‐negative group (*p* = 0.030). Paradoxically, despite their poorer health status, the symptom‐positive group showed lower hospitalization rates (82.1% vs. 88.2%; *p* = 0.010).

**Table 2:** Health outcomes before and after IPTW.

Covariate	Pre‐IPTW (*n* = 418)			Post‐IPTW (*n* = 836)^a^		
	Non‐MHD group (*n* = 216)	MHD group (*n* = 202)	*p*	Non‐MHD group (*n* = 416)	MHD group (*n* = 420)	*p*
Self‐rated health	—	—	0.110	—	—	<0.001
Good	128 (59.3)	104 (51.5)	—	260 (62.5)	199 (47.4)	—
Poor	88 (40.7)	98 (48.5)	—	156 (37.5)	221 (52.6)	—
2‐week morbidity	—	—	0.691	—	—	0.010
No	109 (50.5)	98 (48.5)	—	218 (52.4)	182 (43.3)	—
Yes	107 (48.5)	104 (51.5)	—	198 (47.6)	238 (56.7)	—
Chronic disease prevalence	—	—	0.753	—	—	0.030
No	122 (56.5)	111 (55.0)	—	243 (58.4)	214 (51.0)	—
Yes	94 (43.5)	91 (45.0)	—	173 (41.6)	206 (49.0)	—
Hospitalization rate	—	—	0.327	—	—	0.010
No	27 (12.5)	32 (15.8)	—	49 (11.8)	75 (17.9)	—
Yes	189 (87.5)	170 (84.2)	—	367 (88.2)	345 (82.1)	—

^a^This sample size refers to the weighting of the total population sample size based on IPTW, that is, it is considered a “pseudo‐sample size.”

### 3.4. Healthcare Utilization Inequity Analysis

#### 3.4.1. CI Results

**Figure 2:**
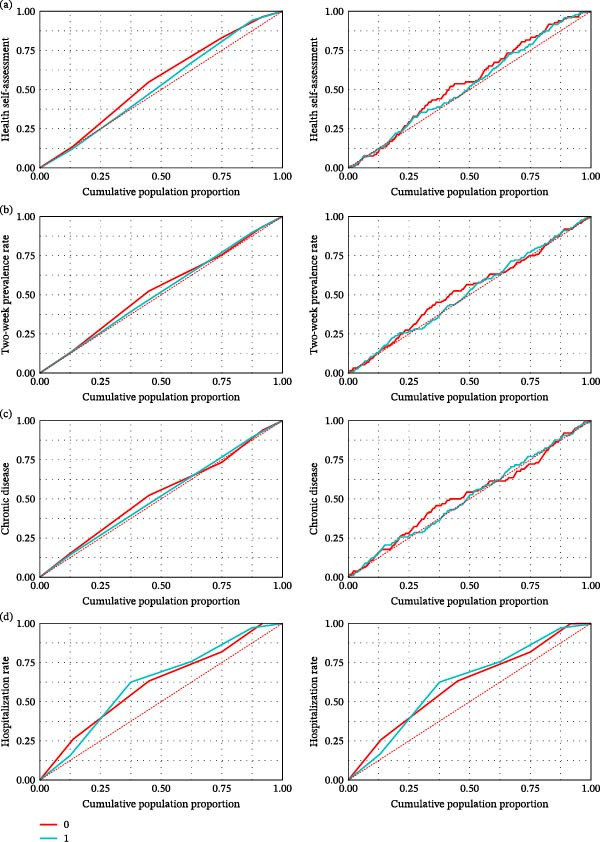
Concentration curves for healthcare utilization outcomes. (a) Self‐rated health. (b) Two‐week morbidity. (c) Chronic disease prevalence. (d) Hospitalization rate.

The CI analysis revealed persistent pro‐rich inequities across all outcomes, though with varying magnitudes between groups (Figure 2, Table 3). The symptom‐positive group consistently showed a lower magnitude of inequity compared to the symptom‐negative group across all measures. For SRH, the CI in the symptom‐positive group was −0.0465, considerably lower than the symptom‐negative group’s −0.0862. This pattern persisted in 2‐week morbidity (symptom‐positive: −0.0188 vs. symptom‐negative: −0.0434) and chronic disease prevalence (symptom‐positive: −0.0138 vs. symptom‐negative: −0.0293). The most pronounced inequities were observed in hospitalization rates, where the symptom‐positive group showed a CI of −0.2379, compared to −0.2656 in the symptom‐negative group.

**Table 3:** Concentration indices for healthcare utilization outcomes.

Variant	Pre‐IPTW		Post‐IPTW	
	Non‐MHD group (*n* = 216)	MHD group (*n* = 202)	Non‐MHD group (*n* = 416)	MHD group (*n* = 420)
Self‐rated health	−0.1108	−0.0496	−0.0862	−0.0465
2‐week morbidity	−0.0567	−0.0262	−0.0434	−0.0188
Chronic disease prevalence	−0.0537	−0.0335	−0.0293	−0.0138
Hospitalization rate	−0.2204	−0.2398	−0.2656	−0.2379

### 3.5. Decomposition Analysis

The decomposition analysis revealed distinct patterns of contributing factors to healthcare utilization inequities in the symptom‐positive group (Table 4). Regarding SRH inequity, drug use history emerged as the primary contributor with a substantial negative effect (−1.1 × 10^1^%, *p* < 0.001), indicating that drug use history significantly exacerbated health inequity, while treatment duration showed a small but significant positive contribution (2.9 × 10^−3^%, *p* = 0.0489), suggesting that an increase in treatment duration somewhat alleviated health inequity. For 2‐week morbidity inequity, regular exercise (3–5 times/week) demonstrated a significant positive contribution (1.4 × 10^0^%, *p* = 0.0099), indicating that regular exercise reduced inequity, while health record establishment showed a marginally significant positive effect (1.4 × 10^0^%, *p* = 0.0507), suggesting that although health record establishment had some alleviating effect on inequity, its impact was not significant.

**Table 4:** Decomposition analysis of healthcare utilization inequities.

Independent variable	Self‐rated health		2‐week morbidity		Chronic disease prevalence		Hospitalization rate	
	Ratio	Contribution rate	Ratio	Contribution rate	Ratio	Contribution rate	Ratio	Contribution rate
Alcohol consumption (no)								
Yes	−4.7 × 10^−2^	−7.5 × 10^−2^	7.6 × 10^−2^	−4.6 × 10^−1^	8.3 × 10^−2^	−3.9 × 10^−1^	7.2 × 10^−2^	−1.5 × 10^0^
Smoking (no)								
Yes	4.2 × 10^−2^	6.8 × 10^−1^	−5.0 × 10^−2^	3.0 × 10^−1^	−5.6 × 10^−2^	2.6 × 10^−1^	1.1 × 10^−1^	−2.2 × 10^0^
Exercise (daily)								
3–5 times per week	4.3 × 10^−2^	6.9 × 10^−1^	−2.3 × 10^−1^ ^∗∗^	1.4 × 10^0^	−8.1 × 10^−2^	3.9 × 10^−1^	6.9 × 10^−2^	−1.4 × 10^0^
1–2 times per week	1.5 × 10^−1^	2.4 × 10^0^	9.0 × 10^−2^	5.5 × 10^−1^	8.1 × 10^−2^	3.8 × 10^−1^	4.8 × 10^−2^	9.7 × 10^−1^
Less than 1 time per week	1.7 × 10^−1^	2.7 × 10^0^	−5.2 × 10^−1^	3.2 × 10^0^	−3.4 × 10^−1^ ^∗∗^	1.6 × 10^0^	1.5 × 10^−1^	3.0 × 10^0^
never	6.2 × 10^−2^	1.0 × 10^0^	−7.6 × 10^−2^	4.6 × 10^−1^	6.5 × 10^−2^	−3.1 × 10^−1^	8.4 × 10^−3^	−1.7 × 10^−1^
Insurance type (employee)								
Inhabitants	2.1 × 10^−2^	3.5 × 10^−1^	−4.0 × 10^−1^	2.5 × 10^0^	−4.7 × 10^−1^	2.2 × 10^0^	5.9 × 10^−2^	−1.2 × 10^0^
Else	−4.0 × 10^−2^	−6.4 × 10^−1^	−4.8 × 10^−1^	2.9 × 10^0^	−5.6 × 10^−1^	2.6 × 10^0^	3.9 × 10^−2^	−7.8 × 10^−1^
Health records (yes)								
No	−1.2 × 10^−1^	−1.9 × 10^0^	−2.3 × 10^−1^+	1.4 × 10^0^	−1.3 × 10^−1^	6.0 × 10^−1^	−1.5 × 10^−1^ ^∗∗∗^	3.1 × 10^0^
Currently unknown	−7.3 × 10^−2^	−1.2 × 10^0^	−1.3 × 10^−1^+	8.0 × 10^−1^	−1.5 × 10^−1^ ^∗^	7.0 × 10^−1^	2.0 × 10^−2^	−3.9 × 10^−1^
Regular medical check‐ups (yes)								
No	1.3 × 10^−1^+	2.1 × 10^0^	1.4 × 10^−1^+	−8.4 × 10^−1^	1.4 × 10^−1^+	−6.6 × 10^−1^	4.6 × 10^−2^	−9.2 × 10^−1^
Route of infection (marital sex)								
Heterosexual sex outside of marriage	−1.7 × 10^−1^	−2.8 × 10^0^	−1.3 × 10^−1^	7.7 × 10^−1^	−1.4 × 10^−1^	6.8 × 10^−1^	6.5 × 10^−2^	−1.3 × 10^0^
Homosexual sex	−1.7 × 10^−1^	−2.7 × 10^0^	1.3 × 10^−1^	−8.1 × 10^−1^	5.5 × 10^−2^	−2.6 × 10^−1^	2.4 × 10^−1^+	−4.7 × 10^0^
Take drugs	−6.5 × 10^−1^ ^∗∗∗^	−1.1 × 10^1^	1.9 × 10^−3^	−1.1 × 10^−2^	2.1 × 10^−1^	−9.8 × 10^−1^	−8.5 × 10^−2^	1.7 × 10^0^
Length of infection	−1.0 × 10^−4^+	−2.2 × 10^−3^	0.0 × 10^0^	−1.0 × 10^−4^	0.0 × 10^0^	1.0 × 10^−4^	−2.0 × 10^−4^	3.2 × 10^−3^
Treatment duration	2.0 × 10^−4^ ^∗^	2.9 × 10^−3^	0.0 × 10^0^	−2.0 × 10^−4^	1.0 × 10^−4^	−3.0 × 10^−4^	1.0 × 10^−4^	−2.9 × 10^−3^
Knowledge of HIV‐related policies (yes)								
No	−7.4 × 10^−2^	−1.2 × 10^0^	3.2 × 10^−2^	−1.9 × 10^−1^	2.7 × 10^−2^	−1.3 × 10^−1^	1.0 × 10^−1^+	−2.1 × 10^0^
Last CD4	−2.0 × 10^−4^	−2.5 × 10^−3^	−2.0 × 10^−4^	9.0 × 10^−4^	−2.0 × 10^−4^	8.0 × 10^−4^	2.0 × 10^−4^+	−3.4 × 10^−3^
Last viral load	4.9 × 10^−2^	7.8 × 10^−1^	3.4 × 10^−2^	−2.1 × 10^−1^	1.1 × 10^−1^	−5.0 × 10^−1^	−7.9 × 10^−2^	1.6 × 10^0^

*Note*: Models were corrected for demographic characteristics gender, age, address, BMI, marital status, work status, years of education, income, and included inverse probability sampling weights. +*p* < 0.1.

^∗^ *p* < 0.05.

^∗∗^ *p* < 0.01.

^∗∗∗^ *p* < 0.001.

In terms of chronic disease inequity, limited physical activity (less than once per week) emerged as a significant contributor (1.6 × 10^0^%, *p* = 0.0010), indicating that a lack of physical activity significantly exacerbated chronic disease inequity, alongside health record establishment (7.0 × 10^−1^%, *p* = 0.0458), suggesting that health record establishment somewhat alleviated chronic disease inequity. The analysis of hospitalization inequity identified health record establishment as the strongest positive contributor (3.1 × 10^0^%, *p* < 0.001), indicating that it significantly alleviated hospitalization inequity, while sexual orientation showed a marginally significant negative association (−4.7 × 10^0^%, *p* = 0.0810), suggesting that the impact of sexual orientation somewhat alleviated hospitalization inequity, but the impact was relatively small.

## 4. Discussion

Using IPTW methodology, this study examined healthcare utilization inequities among PLHIV with and without mental health disorders in China. The findings revealed significant disparities in both health outcomes and healthcare utilization patterns, with three key observations: (1) PLHIV with mental health disorders demonstrated significantly poorer health outcomes; (2) despite worse health status, this group showed lower hospitalization rates; and (3) healthcare utilization inequities exhibited distinct patterns between groups, with particularly pronounced disparities in hospitalization rates.

The poorer health outcomes observed among PLHIV with mental health disorders align with previous research in resource‐limited settings [26]. This association likely reflects multiple underlying mechanisms. Psychological distress associated with mental health disorders can lead to immune system dysregulation [27], which makes it more prone to comorbidities, such as chronic diseases, digestive system disease control [28], and somatization symptoms [29], potentially accelerating HIV disease progression [30]. Mental health conditions may also interfere with medication adherence [31], as depression and anxiety symptoms can impair motivation and daily functioning. Furthermore, the stigma associated with both HIV and mental health conditions may create barriers to accessing healthcare services and individuals may perceive accessing health services as potentially leading to more threatening outcomes [6], such as the fear of disclosing their infection status and distrust of, or fear of, healthcare providers [32], leading to delayed treatment and poorer health outcomes.

A striking finding was the lower hospitalization rate among PLHIV with mental health disorders despite their worse health status. This paradox suggests significant barriers to healthcare access, consistent with previous studies documenting reduced healthcare utilization among individuals with comorbid conditions [33]. Several interrelated factors may explain this pattern. First, the unique context of HIV care may be exacerbating mental health–related care avoidance; for example, anxiety can foster healthcare disengagement, while depressive symptoms may reduce outpatient follow‐up due to hopelessness or diminished motivation [34]. Second, structural barriers persist, including fragmented care systems that lack integrated HIV and mental health services [35] and financial constraints exacerbated by mental health–related employment instability [36, 37]. Finally, cognitive impairments associated with depression and anxiety can further hinder individuals’ ability to recognize health needs and navigate care systems [38].

The CI analysis revealed an unexpected pattern of healthcare utilization inequities. While both groups showed pro‐rich inequities, the magnitude was consistently smaller in the symptom‐positive group across all outcomes. This finding contrasts with previous research suggesting that mental health conditions typically amplify healthcare inequities [39–41]. Several context‐specific factors in China’s healthcare system may explain this observation.

China’s comprehensive HIV care policy, particularly the “Four Frees and One Care” program, provides free antiretroviral treatment and related services [42]. This policy significantly reduces financial barriers to HIV care, especially benefiting socioeconomically disadvantaged individuals with mental health disorders. Additionally, China’s mental health reform and its integration into primary healthcare services have emphasized community‐based mental health services and their inclusion in the basic public health service package [43]. The positive effect of health record establishment on reducing inequities (contribution: 3.1 × 10^0^%, *p* < 0.001) suggests the success of these integrated approaches.

The extensive network of CDC facilities in China, coupled with the hierarchical medical system, provides regular follow‐up and standardized management for PLHIV [43]. This systematic tracking and management system may be particularly effective in maintaining healthcare engagement among those with mental health disorders, regardless of their economic status. However, it is important to note that the smaller magnitude of inequity in the symptom‐positive group does not necessarily indicate better healthcare access. Rather, it might reflect a more uniformly challenging healthcare environment for this group, as evidenced by their lower overall hospitalization rates despite worse health status.

These findings highlight both the successes and remaining challenges in China’s approach to HIV and mental healthcare integration. While existing policies appear to help mitigate income‐related healthcare inequities, the lower overall healthcare utilization among those with mental health disorders suggests a need for more targeted interventions addressing nonfinancial barriers to care. The decomposition analysis identified several key contributors to healthcare utilization inequities. Drug use history emerged as a significant factor (−10.51%, *p* < 0.001), highlighting the complex interplay between substance use, mental health, and healthcare access [44, 45]. Physical activity patterns also played a notable role, with regular exercise showing protective effects against healthcare inequities. These findings suggest that lifestyle interventions might help reduce healthcare utilization disparities, though the causal direction of these relationships requires further investigation.

Our findings have important implications for healthcare policy and service delivery. First, they highlight the need for integrated HIV and mental health services, as recommended by recent WHO guidelines [9, 46, 47]. Such integration could help address the complex needs of PLHIV with mental health disorders and reduce barriers to care. Second, the positive impact of health record establishment suggests that strengthening primary care infrastructure and systematic healthcare management could help reduce utilization inequities. Third, the identified role of lifestyle factors indicates potential targets for intervention programs.

This study has several limitations that should be considered. First, the assessment of mental health was based on screening tools (PHQ‐9 and GAD‐7) rather than clinical diagnostic interviews, which limits the ability to infer clinical diagnoses of depression or anxiety disorders. Second, while the IPTW methodology effectively balanced observed confounders, unmeasured confounding may persist, particularly regarding psychological factors and social support networks that could influence both mental health and healthcare utilization. Third, “symptom‐positive,” as screening positive on either the PHQ‐9 or GAD‐7, may mask potential heterogeneity between depressive and anxiety symptoms. Our current definition does not distinguish between subgroups with “pure depression,” “pure anxiety,” and “comorbid” conditions. Given the sample size (*N* = 418), further stratification into these subgroups would result in insufficient statistical power for some categories, potentially compromising the stability and interpretability of the results. Future research with larger sample sizes is warranted to conduct subgroup analyses examining the differential associations of pure depression, pure anxiety, and comorbid states with healthcare utilization inequities among PLHIV.

This study provides robust evidence of significant disparities in health outcomes and healthcare utilization among PLHIV with mental health disorders in China. The findings highlight the complex interplay between the mental health status, healthcare access, and socioeconomic factors in determining healthcare utilization patterns. Future interventions should focus on integrating mental health services into HIV care, strengthening systematic healthcare management, and addressing lifestyle factors that contribute to healthcare inequities. Such comprehensive approaches will be crucial for promoting more equitable health outcomes in this vulnerable population.

## Funding

This study was funded by the Ministry of Education Humanities and Social Science Project (Grant 24XJCZH002).

## Disclosure

After using ChatGPT, the authors reviewed and edited the content as needed and take full responsibility for the content of the publication.

## Conflicts of Interest

The authors declare no conflicts of interest.

## Data Availability

Research data are not shared.
